# Exploring the Association Between DTC Obesity-Related Gene Polymorphisms and Obesity Risk Factors in Koreans: Focus on *BDNF*

**DOI:** 10.3390/nu18040655

**Published:** 2026-02-16

**Authors:** Jiha Kim, Soyoun Lee, Myoungsook Lee

**Affiliations:** 1 Department of Food and Nutrition, Sungshin Women’s University, Dobong-ro 76ga-gil 55, Gangbuk-gu, Seoul 01133, Republic of Korea; jihak1209@gmail.com (J.K.); special8616@gmail.com (S.L.); 2 GenDietCare Co., Ltd., Building B-108, Sungshin Women’s University, Dobong-ro 76ga-gil 55, Gangbuk-gu, Seoul 01133, Republic of Korea

**Keywords:** precision nutrition, DTC genetic testing, *BDNF*, obesity phenotypes, resting metabolic rate (RMR), dietary intake

## Abstract

Background/Objectives: Among more than 300 candidate genes for obesity, *FTO*, *MC4R*, and *BDNF* have been approved for DTC genetic testing. However, population-specific evidence supporting their relevance to obesity-related phenotypes in Koreans remains limited. Methods: A total of 231 healthy adults aged 19–64 years were recruited between March and May 2024. Anthropometric and clinical measurements, genotyping, dietary intake, and questionnaires on socioeconomic status, family history, and lifestyle behaviors were obtained. Associations between genotypes and obesity-related phenotypes were evaluated using ANOVA and ANCOVA, multivariable-adjusted models and multicollinearity analysis-based stepwise regression. Results: In Koreans, MAFs for *FTO (3 SNPs), MC4R rs17782313* and *BDNF rs6265* were 13–16%, 27.1% and 47.4%, respectively. OB frequency (%) differed significantly between *BDNF* GG and A allele carriers (*p* < 0.05). Compared to GG, *BDNF* A allele carriers showed higher WHR, ALT, HbA1c and sodium intake (*p* < 0.05). *BDNF* A allele carriers were observed to have higher drinking frequency and elevated ALT levels. Significant genotype–obesity interactions were identified for RMR/BW status, dietary fiber, Vit E, folate, P, K, cholesterol, and PUFA (*p* < 0.05). Among A allele carriers, OB-related indicators (BMI, RMR, WHR) were independently associated with age, sex, RMR, SBP, ALT, leptin, and dietary intakes of Vit A and sugars. Conclusions: These findings support the relevance of *BDNF* rs6265 in obesity phenotypes among Korean adults and provide Korean-specific evidence for genotype-based nutrition strategies. Given the cross-sectional study, the interpretation of personalized nutrition approaches for genetic risk carriers should be made with caution.

## 1. Introduction

Obesity is defined as a condition characterized by excessive fat accumulation in the body and is a major risk factor that increases mortality associated with various diseases, including metabolic syndrome, cardiovascular and cerebrovascular diseases, diabetes, dyslipidemia, and cancer [1,2,3,4]. In Korea, the prevalence of adult obesity has also shown a persistent upward trend over the past decade. In 2020, largely due to the impact of the COVID-19 pandemic, prevalence rose to 38.3%, followed by a slight decline, stabilizing at 37.2% in 2023, which was consistent with the previous year [5]. This rising prevalence of obesity is a social and structural phenomenon intertwined with demographic shifts, economic development, and lifestyle changes, and thus cannot be resolved solely through individual efforts. Accordingly, systematic and coordinated policy interventions by multiple government sectors are required [6].

It is well established that obesity arises from a complex interplay of dietary patterns, lifestyle, and environmental factors. Furthermore, obesity exhibits unique features across ethnicities, sexes, and life stages, underscoring the importance of genetic research to identify relevant determinants [7]. Although approximately 300 candidate genes for obesity have been identified, strong ethnic specificity hinders the selection of universally applicable genes, making population-specific genetic studies essential [8,9]. With growing national attention to obesity prevention and management, the demand for direct-to-consumer (DTC) genetic testing has also increased [10]. In 2024, 190 DTC items have been officially approved in Korea; however, according to the Ministry of Health and Welfare notification (No. 2023-300), only one obesity-related item—body mass index (BMI)—is currently permitted for DTC genetic testing [11]. Moreover, only three candidate genes have been linked to BMI in the context of Korea: fat mass and obesity-associated (*FTO*), melanocortin-4 receptor (*MC4R*), and brain-derived neurotrophic factor (*BDNF*) [12].

We compared minor allele frequencies (MAFs) for *FTO*, *MC4R*, and *BDNF* identified in this study with those reported in prior studies and publicly available data from the National Institutes of Health (NIH) dbSNP database (Supplementary Materials, Table S1) [13,14,15,16,17,18,19]. In this study, the MAFs for *FTO* and *MC4R rs17782313* were 13.0–16.2% and 27.1%, respectively. They were consistent with previous Korean studies and data from the Korean Genome Project (KGP 4K), the Korean Reference Genome Database (KRGDB), and East Asian data from the National Center for Biotechnology Information (NCBI). However, MAF of *BDNF rs6265* was 47.4%, which differed from some previous reports but was comparable to KoGES data and NCBI East Asian reference values. Since SNP-specific MAFs vary across populations (e.g., European, African, Asian, and Latin American), the establishment of DTC genetic testing should be based on population-specific scientific evidence. In international cases, a broader range of obesity-related traits are examined, including not only BMI but also weight gain, weight management difficulties, metabolic syndrome, appetite regulation, satiety, and responses to specific dietary patterns such as the Mediterranean diet, low-fat diets, and low-carbohydrate diets. Research to identify novel phenotypes with strong associations to obesity-related genes is actively ongoing and has already been applied to healthcare industries abroad [20]. By contrast, in Korea, the phenotypes associated with DTC obesity genes remain limited to BMI, which constrains the exploration of diverse obesity manifestations. Given these limitations, further foundational research to expand the phenotypic spectrum of obesity in Koreans is urgently needed. In this study, we observed differences in MAF compared with Western populations. Notably, analysis of the relationship between MAF and obesity status identified significant associations for *BDNF*, which was thus selected as the primary focus for subsequent analyses.

Precision nutrition is a personalized dietary strategy that integrates genetic variation with anthropometric, metabolic, and behavioral data to tailor nutritional interventions aimed at improving metabolic efficiency and reducing disease risk. Genomic information reflecting individual variability constitutes a fundamental pillar of precision nutrition, and growing consumer interest in DTC genetic testing has rapidly accelerated its commercialization. However, regulatory approval from governments should be limited to genetic tests supported by robust scientific evidence derived from Korean populations. Moreover, clear guidelines remain insufficient with respect to the effective use of DTC genetic information for disease prevention strategies and individualized management approaches. Therefore, we hypothesized that, despite being approved for DTC genetic testing, some obesity-related genes may lack sufficient or population-specific scientific evidence in Koreans. Accordingly, the purpose of this study is to validate scientific evidence supporting obesity-related gene DTC genetic testing and to assess the appropriateness of their use in the Korean DTC context. Furthermore, the ultimate objective of this study is to contribute to the development of gene-based precision nutrition services for weight control tailored to Korean populations, with a focus on genetically supported targets.

## 2. Materials and Methods

### 2.1. Study Design and Population

**Figure 1:**
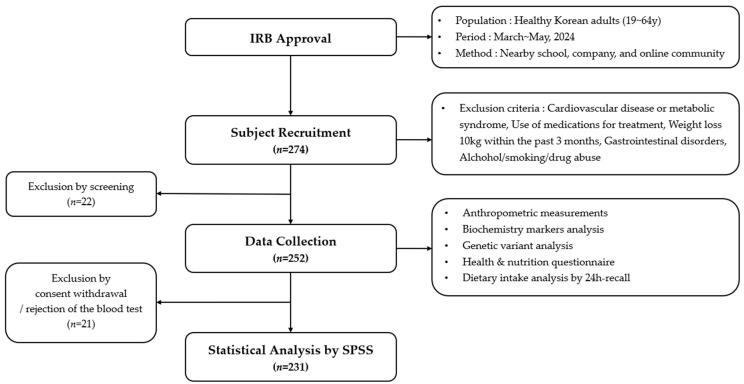
Flowchart of study design.

This study recruited healthy Korean adults aged 19–64 years between March and May 2024 through public advertisements posted at nearby universities, workplaces, and online communities. After screening based on predefined exclusion criteria, 252 individuals consented to participate. Following the exclusion of 21 participants who withdrew consent or declined blood sampling, a total of 231 participants (female; *n* = 200, male; *n* = 31) were included in the final analysis (Figure 1).

At the 1st visit, after all participants received an explanation of the study, they provided written informed consent. At this time, the questionnaires and dietary record forms were distributed, and participants were instructed to complete them in advance and bring the completed materials to the second visit. At the 2nd visit, anthropometric measurements and blood collection for testing were performed, and previously distributed questionnaires and dietary assessments were verified. The study protocol and procedures were reviewed and approved by the Institutional Review Board (IRB) of Sungshin Women’s University (approval no. SSWUIRB-2024-008).

### 2.2. Data Collection

#### 2.2.1. Anthropometric Measurements

All measurements were obtained after an overnight fast of at least 8 h. Height (Ht), weight (Wt), waist circumference (WC), hip circumference (HC), and blood pressure—systolic (SBP) and diastolic (DBP)—were measured (Omron Healthcare Korea Co., Ltd., Seoul, Republic of Korea). Body mass index (BMI), skeletal muscle mass (SMM), and fat mass (FM) were assessed using bioelectrical impedance analysis (BIA) with the InBody 770 device (InBody Co., Ltd., Seoul, Republic of Korea). Waist-to-hip ratio (WHR) was calculated as WC (cm) divided by HC (cm). Predicted resting metabolic rate (RMR) was calculated using the Harris–Benedict (H&B) equations (men: 66.5 + 13.8 × Wt + 5.0 × Ht − 6.8 × A; women: 655.1 + 9.6 × Wt + 1.9 × Ht − 4.7 × A, where A denotes age [21]). Obesity status was defined using the World Health Organization Asia–Pacific criteria: BMI < 23 kg/m^2^ as normal, 23.0–24.9 kg/m^2^ as overweight, and ≥25.0 kg/m^2^ as obese. For analysis, participants were categorized into a non-obese group (non-OB) and an obese group (OB).

#### 2.2.2. Clinical Markers

After an overnight fast of at least 8 h, venous blood was drawn by a nurse into EDTA tubes (whole blood) and SST tubes (serum). Samples were immediately mixed and centrifuged, then stored at −80 °C until analysis. Using whole blood and serum, the following analytes were assessed: fasting blood glucose (FBS), hemoglobin A1c (HbA1c), triglycerides (TG), total cholesterol (TC), high-density lipoprotein cholesterol (HDLc), low-density lipoprotein cholesterol (LDLc), aspartate aminotransferase (AST), alanine aminotransferase (ALT), and leptin. HbA1c concentrations in whole blood were determined by enzyme colorimetric assay using a Cobas c513 Chemistry Analyzer (Roche Diagnostics, Mannheim, Germany). Serum FBS, AST, ALT, TG, TC, HDLc, and LDLc were analyzed by enzyme colorimetric assays on a Cobas c502 Chemistry Analyzer (Roche Diagnostics, Germany). Leptin concentrations were measured using commercial ELISA kits (Millipore EZHL-80SK, Human Leptin “Dual Range” ELISA; Sigma-Aldrich, Seoul, Republic of Korea). All analyses were performed at SCL Healthcare (Seoul Clinical Laboratories, Seoul, Republic of Korea) in accordance with the manufacturers’ protocols.

Clinical reference ranges and cut-off values were defined according to established clinical guidelines. The upper limits of AST and ALT levels were provided by SCL Healthcare (Seoul Clinical Laboratories, Seoul, Republic of Korea), as AST ≤ 40 IU/L for men and ≤32 IU/L for women, and ALT ≤ 41 IU/L for men and ≤33 IU/L for women. TG levels were classified as normal (<150 mg/dL), borderline-high (150–199 mg/dL), and high (≥200 mg/dL), and TC levels were considered desirable when <200 mg/dL, based on the National Cholesterol Education Program Adult Treatment Panel III (NCEP ATP III) guidelines. HDLc levels were defined as low when <40 mg/dL for men and <50 mg/dL for women, and LDLc levels were classified as optimal when <100 mg/dL, also according to NCEP ATP III. HbA1c levels < 5.7% were considered normal according to the American Diabetes Association (ADA) criteria.

#### 2.2.3. General Questionnaires and Dietary Intake Assessment

On the 2nd visit, the previously distributed questionnaires and dietary records were retrieved. The questionnaires were reconstructed from the Korea National Health and Nutrition Examination Survey on education and income levels, family history of disease, lifestyle, physical activity, and dietary habits.

All dietary intake was assessed using three non-consecutive 24 h dietary recalls, consisting of two weekdays and one weekend. The trained dietitians verified the records through face-to-face interviews to improve the accuracy of portion size estimation using standardized measuring tools and food models, and the research team conducted cross-checks. Nutrient intakes were quantitatively analyzed based on the collected dietary records using CAN-Pro 6.0 Software (The Korean Nutrition Society; KNS, Seoul, Republic of Korea). Standard reference values for food and nutrient intake were based on the 2020 Dietary Reference Intakes for Koreans from the KNS.

### 2.3. Genotyping Analysis

Genomic DNA extraction and genotyping by TaqMan and SNaPShot assays were performed by DNA Link Inc. (Seoul, Republic of Korea). Genomic DNA was extracted from whole blood using the QuickGene DNA Whole Blood Kit S (Kurabo, Osaka, Japan) following the manufacturer’s instructions.

*FTO* (*rs9939609*) was genotyped using a TaqMan assay fluorescence 5′ nuclease assay (Applied Biosystems, Foster City, CA, USA, while *FTO* (*rs9939973*, *rs8050136*), *BDNF* (*rs6265*), and *MC4R* (*rs17782313*) were analyzed using the SNaPShot Multiplex Kit (Applied Biosystems, Foster City, CA, USA). Methods details are summarized in Supplementary Materials, Table S2. Genotypes were called using GeneMapper software (version 4.0, Applied Biosystems).

### 2.4. Statistical Analyses

Continuous variables, including anthropometric indices, clinical parameters, and dietary data were analyzed using independent samples *t*-tests, one-way analysis of variance (one-way ANOVA), and two-way ANOVA, and are presented as mean ± standard deviation (SD). Student’s *t*-test was used under the assumption of homogeneity of variance; when homoscedasticity was violated, Welch’s *t*-test was applied. Categorical variables, such as general characteristics, health behaviors, dietary characteristics, and genotypes were summarized as frequency (*n*) and percentage (%) and compared between groups using the chi-square test, with *p*-values reported from Pearson’s chi-square test or Fisher’s exact test as appropriate. Because the frequency of the AA genotype for *BDNF* was low, carriers of the A allele (GA + AA) were combined into a single group to ensure statistical power. In genotyping, there were no samples excluded during the analysis due to failure to meet quality criteria (success rate: 100%). No DNA quality control was assessed based on the 260/280 absorbance ratio (range: 1.5–2.2), total DNA yield (≥0.05 μg), and minimal concentration (≥10 ng/μL), and all samples met these quality thresholds. The Hardy–Weinberg equilibrium (HWE) was evaluated for each SNP in the control group using a chi-square test, and all SNPs conformed to HWE (*p* ≥ 0.05).

To compare differences across four groups defined by genotype (GG vs. AG + AA) and obesity status (non-OB vs. OB), such as GG/non-OB, GG/OB, AG + AA/non-OB, and AG + AA/OB, one-way ANOVA was performed, followed by Scheffé’s post hoc test. Two-way ANOVA was used to examine interactions between genotype and obesity status. Analysis of covariance (ANCOVA) was conducted to compare adjusted group means after controlling for sex, age, and daily energy intake. To identify determinants influencing obesity-related indices associated with the *BDNF* genotype, stepwise linear regression analyses were performed. A *post hoc* power analysis using G*Power (version 3.1.9) was conducted to evaluate whether the sample size was adequate to detect the observed SNP effects. It is based on linear multiple regression (fixed model, R^2^ increase), incorporating the observed effect size (f^2^), total sample size, number of predictors, and a significance level of α = 0.05. Model fit was evaluated using the coefficient of determination (R^2^) and adjusted R^2^ (adj. R^2^). Dietary nutrient variables were energy-adjusted using the residual method based on total energy intake. Pearson’s correlation analysis was performed among independent variables to assess potential collinearity prior to regression analyses. To control for multiple testing, *p*-values were adjusted using the Benjamini–Hochberg false discovery rate (FDR) procedure. Multicollinearity among independent variables was assessed by examining variance inflation factors (VIF) and tolerance values, with VIF < 10 and tolerance > 0.1 indicating no serious multicollinearity. Pearson’s correlation analysis was additionally performed to examine bivariate relationships among predictors prior to regression analyses. All statistical analyses were conducted using SPSS version 29.0 (IBM SPSS Statistics; SPSS Inc., Chicago, IL, USA). Statistical significance was set at *p* < 0.05 (*), *p* < 0.01 (**), *p* < 0.001 (***).

## 3. Results

### 3.1. General Characteristics of Participants

Health-related characteristics, including anthropometrics (Ht, Wt, BMI, RMR, RMR/BW, WC, SBP, DBP) and clinical parameters (AST, ALT, FBS, HbA1c, TG, TC, HDLc, LDLc), were analyzed by obesity status and by sex. Group differences in questionnaire items (sociodemographics, family history, health-related behaviors, and dietary habits) by obesity status and by sex were evaluated using the chi-square test.

Comparisons of anthropometrics between the non-OB (*n* = 103) and OB (*n* = 128) groups (Table 1) showed that the OB group had significantly higher values for all indices (BMI, Wt, RMR, RMR/BW, WC, WHR) than the non-OB group. Notably, RMR remained significantly higher in the OB group both before and after adjustment, whereas RMR/BW tended to be lower in the OB group, indicating that metabolic rate per unit body weight decreases as total body weight increases. For clinical parameters, both SBP and DBP were significantly higher in the OB group than in the non-OB group; however, these differences diminished or disappeared after adjustment for age and sex. Hepatic injury markers AST and ALT were significantly higher in the OB group, while the AST/ALT ratio was significantly higher in the non-OB group, both before and after adjustment. HbA1c, a long-term glycemic marker, was significantly higher in the OB group. Among blood lipid indices, TG, TC, and LDLc were significantly higher in the OB group, whereas HDLc was significantly lower. In addition, serum leptin concentrations were significantly higher in the OB group than in the non-OB group.

When anthropometrics and clinical parameters were compared by sex (women, *n* = 200; men, *n* = 31), most variables differed significantly (Table 1). Men exhibited significantly higher values than women—both before and after adjustment—for most body composition indices (BMI, height, weight, RMR, WC, WHR). In particular, mean RMR was markedly higher in men (1796.06 kcal), while RMR/BW was lower in men than in women.

Overall, men showed higher clinical values than women, notably for blood pressure (SBP, DBP), hepatic markers (AST, ALT), glycemic indices (FBS, HbA1c), and lipid parameters (TG, TC, LDLc). Indices that remained significantly higher in men both before and after adjustment included SBP, DBP, AST, ALT, FBS, HbA1c, and TG. By contrast, women showed higher AST/ALT ratios (1.54), higher HDLc, and higher leptin. In particular, serum leptin, a hormone secreted by adipocyte, was approximately two-fold higher in women (23.14 ng/mL) than in men (11.27 ng/mL), paralleling the pattern observed by obesity status wherein OB (26.87 ng/mL) exceeded non-OB (14.94 ng/mL) by roughly twofold.

Most sociodemographic, dietary, and health behavioral variables did not differ significantly according to obesity status or sex, except for a higher prevalence of family history of obesity in the obese group and several sex-specific differences in smoking, alcohol use, breakfast skipping, and eating-out frequency. Detailed distributions and corresponding *p*-values for all questionnaire items are presented in Supplementary Materials, Table S3.

### 3.2. Comparisons by Genotype

#### 3.2.1. MAF, Obesity Frequency (%), and BDNF Levels by Genotypes

Genotype distributions by polymorphism were compared between the non-OB and OB groups to assess differences in obesity frequency across genotypes (wild, heterozygous, and mutant; or wild and H + M). For FTO, the minor genotype was rare across all three SNPs (rs9939609, rs9939973, rs8050136: 1.3%, 3.1%, and 1.3%, respectively), with most participants clustering in the wild and heterozygous categories; distributions by obesity status were not significant. Likewise, MC4R rs17782313 showed no significant between-group difference in genotype distribution and exhibited similar patterns in non-OB and OB (Table 2).

**Figure 2:**
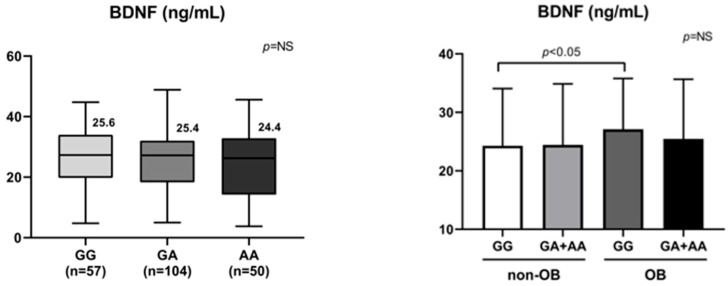
Protein levels of BDNF (ng/mL) according to different genotypes and the changes in BDNF proteins in OB compared to non-OB according to different genotypes. NS denotes no statistically significant difference.

For BDNF rs6265, overall differences were not statistically significant in the three-level breakdown, although the heterozygous proportion was relatively higher in OB. We focused on BDNF because it showed a high MAF in Koreans and a significantly greater OB frequency in individuals with GA + AA carriers compared with the GG type. These characteristics justified the use of a dominant genetic model for comparison (Table 2). Blood BDNF concentrations were slightly lower in individuals carrying GA or mutant allele compared with the wild genotype, although the differences did not reach statistical significance. This pattern suggests a potentially reduced biological sensitivity or functional response of BDNF among A allele carriers. Interestingly, BDNF protein levels were similar between GG and GA + AA carriers in the non-OB group; however, in the OB group, BDNF levels were significantly elevated in GG carriers compared with the GA + AA (Figure 2). This may indicate a compensatory increase in circulating BDNF in response to obesity to suppress food intake in wild carriers, whereas it appears to be blunted in A allele carriers.

#### 3.2.2. Risk Indicators by BDNF Genotypes

**Figure 3:**
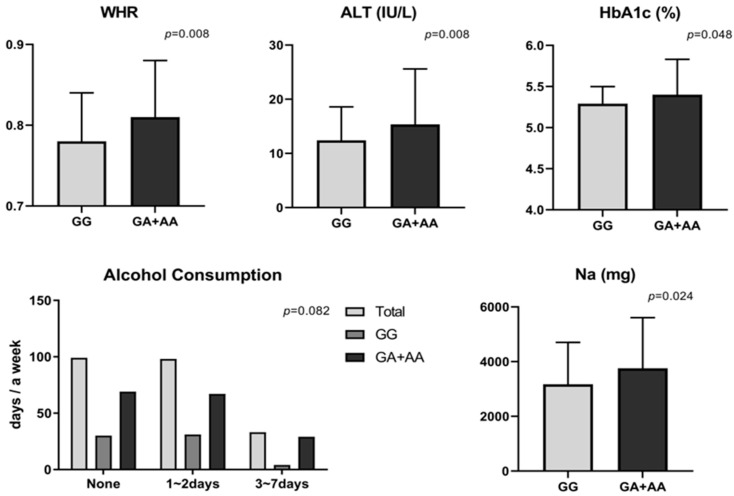
Significant differences in the risk factors (anthropometrics, clinics, and diets) between *BDNF rs6265* GG homozygotes and A allele carriers (GA + AA genotypes). *p*-values were calculated using Student’s *t*-test or Welch’s *t*-test, as appropriate, whereas alcohol consumption was analyzed using the chi-square test.

When anthropometric indices were compared between GG (*n* = 65) and GA + AA (*n* = 166), only WHR differed significantly. As an index of abdominal obesity, WHR was 0.78 in wild and 0.81 in GA + AA carriers, indicating a higher value in GA + AA; however, the significance disappeared after adjustment. Since the additional BIA measures for body composition were not significant according to *BDNF* gene variation, they could not be considered for new phenotypes. Among metabolic clinical parameters, the A allele carriers showed higher values for DBP (*p* = 0.077; trend), hepatic enzymes (AST, ALT), and HbA1c than wild carriers in unadjusted analyses. However, most of these pre-adjustment differences lost significance after adjustment (Figure 3).

Across *BDNF rs6265* genotypes, most questionnaire variables did not differ, including sociodemographic factors, family histories, health behaviors, and exercise patterns. Interestingly, alcohol consumption showed only a weak tendency toward higher drinking frequency in A allele carriers than in GG type (χ^2^ = 4.993, *p* = 0.082), which was consistent with their slightly higher hepatic enzymes (AST and ALT), HbA1c, and WHR, suggesting a possible link between the variant and an adverse metabolic profile (Figure 3). Detailed values are presented in Supplementary Materials, Table S3.

Dietary analyses by *BDNF* genotype revealed no significant differences for most nutrients between GG and GA + AA genotypes. However, sodium intake was significantly higher in A allele carriers (Figure 3), suggesting a possible tendency toward high sodium consumption among *BDNF* A allele carriers. Although potassium and the Na/K ratio did not differ significantly, both were numerically higher in A allele carriers. In practical terms, individuals with the *BDNF* A allele may benefit from sodium reduction and alcohol abstinence, as failure to modify these behaviors could plausibly contribute to adverse shifts in obesity phenotypes, including elevations in hepatic injury markers, glycemic indices, and central adiposity (e.g., WC/WHR).

### 3.3. Interaction Effects Between Genotype and Obesity

To evaluate interaction effects between genotype (GG vs. GA + AA) and obesity status (non-OB vs. OB), two-way ANOVA was performed. For most anthropometric and clinical indicators, no significant interaction between genotype and obesity status was observed (Table 3). However, a significant interaction effect was detected for RMR/BW before adjustment, suggesting that energy metabolism efficiency may differ according to *BDNF* genotype even within the OB group. After adjusting for sex and age, this interaction effect was no longer significant because sex and age, key determinants of RMR, may confound the relationship between *BDNF* genotype and obesity.

However, dietary fiber, Vit E, folate, P, K, Mg, cholesterol, and PUFA significantly interacted with genotype and obesity (Table 3). After controlling for sex, age, and total energy intake, fibers, Vit E, folate, and cholesterol intakes were higher in OB compared to non-OB in A allele carriers. Since the energy intake was higher in OB than non-OB in A allele carriers and it was higher in non-OB than OB in GG types, the all-significant dietary indicators largely followed this genotype-specific energy intake pattern. Obesity acted as a strong confounding factor in the interaction between genotypes and obesity. On the other hand, sodium intake tended to be higher in A allele carriers than in GG type, and higher in OB than non-OB subjects. Although it was not significant because of high SDs, it suggests that sodium reduction may benefit A allele carriers with OB.

### 3.4. Correlations Between Increased Obesity Risk and BDNF Genotype

Stepwise linear regression analysis was conducted to identify variables contributing to obesity risk in association with *BDNF* variants (Table 4). Various dependent variables (e.g., BMI, RMR, WHR) were examined, with all nutrient variables adjusted for energy intake. Pearson’s correlation analysis showed no strong correlations among independent variables (Supplementary Materials, Table S4), and multicollinearity was further excluded based on tolerance and VIF values (Supplementary Materials, Table S5). The *post hoc* power analysis indicated that the regression model had sufficient statistical power (power ≈ 1.00) to detect the observed associations between the *BDNF* rs6265 genotype and obesity phenotypes (BMI, RMR, WHR), suggesting that the sample size was adequate for identifying moderate-to-large genetic effects in this study.

Compared with GG carriers, *BDNF* AG + AA carriers had significantly higher BMI, RMR, and WHR, even when accounting for gender difference. Among metabolic factors, ALT and leptin were positively associated with BMI and RMR, whereas RMR/BW and HDLc were negatively associated with BMI and WHR among *BDNF* AG + AA carriers, respectively. In the regression model for RMR, WHR, SBP, ALT, and leptin were all positively associated, indicating that body fat and metabolic markers contribute to increased energy expenditure. The *BDNF* A allele carriers were positively associated with WHR, suggesting a link between *BDNF* and abdominal fat distribution. RMR/BW was inversely associated with WHR, suggesting that higher metabolic efficiency reduces abdominal adiposity. AST and Vit A intake showed positive associations with WHR, while sugar intake was negatively associated but did not reach significance.

After Benjamini–Hochberg FDR correction, all predictors in the RMR model remained statistically significant associations of the BDNF genotype. However, sugar intake did not survive in the WHR model, and BDNF, WHR, and HDLc variables in the BMI model were less significant than before FDR correction.

## 4. Discussion

Identifying obesity susceptibility genes specific to Koreans is not only scientifically valuable but also crucial for advancing the DTC genetic testing industry. However, domestic studies focused on Koreans have often reported weak or inconsistent associations between DTC OB genes and obesity. Compounding this issue, regulatory authorities should recognize that candidate obesity-related genes recommended by the industry are awaiting DTC approval despite the lack of sufficient Korean-specific evidence [22,23,24]. In this study, *BDNF* was selected as a primary target for DTC testing, because it showed relatively high MAFs (40%) in Koreans and high prevalence of obesity in risk genotype carriers. We proposed precision nutrition strategies to help BDNF risk carriers manage various obesity-related phenotypes based on responsive clinical indicators and dietary references. In contrast, current evidence is insufficient to support the use of *FTO* and *MC4R* genes for DTC testing in the Korean population, and additional evidence from well-designed intervention and longitudinal studies are needed.

The *BDNF* gene encodes a neurotrophin that plays essential roles in neuronal survival, differentiation, synaptic plasticity, learning and memory, and the maintenance of energy homeostasis [25,26]. *BDNF* polymorphisms act as important biomarkers not only for obesity but also for various metabolic and neuropsychiatric disorders, including depression, Alzheimer’s disease, anorexia nervosa, and bulimia nervosa [27]. Within the context of energy balance, *BDNF* contributes to both satiety induction and increased energy expenditure. Multiple GWAS have demonstrated hypothalamic *BDNF* suppresses appetite and increases physical activity, thereby contributing to energy expenditure [28]. Leptin, secreted by adipocytes after food intake, activates the POMC (pro-opiomelanocortin)–α-MSH (α-melanocyte stimulating hormone)–MC4R (melanocortin-4 receptor) pathway, which in turn induces *BDNF* expression [29].

The association between *BDNF* polymorphisms and obesity has been extensively studied across various populations. Although the *BDNF* wild genotype (GG, Val/Val) is considered the reference, it was not the most prevalent in this study: GG 28.1%, GA 48.9%, AA 22.9%. This pattern differs markedly from the Boston Puerto Rican Study (GG 73.2%, GA 49.8%, AA 1.9%) and the Romanian Study (GG 65.5%,GA 30.5%, AA 4.0%) [30,31]; however, it is similar to the Taiwan Biobank Study (GG 25.1%, GA 50.1%, AA 24.7%) [32]. Regarding associations with obesity, Wu et al. reported that *BDNF* variants increase obesity risk with higher odds ratios in Asians than in Europeans [33]. Hong et al. found consistent associations between the Met allele and BMI across three large Korean epidemiologic cohorts, supporting heightened genetic sensitivity in Asian populations [19]. Collectively, these results indicate that *BDNF* polymorphism frequencies vary markedly by ethnicity; the relatively high Met allele frequency in Asians may help explain population differences in obesity risk. Regarding associations with obesity by gender differences, Boston Puerto Rican data showed lower BMI among women with wild, but higher BMI among men with wild, indicating sex-specific, opposing trends [31]. Beckers et al. reported a higher Met allele frequency among obese women, suggesting possible female-specific contributions to obesity risk [34]. Thus, in European ancestry populations, *BDNF* obesity associations often differ by sex, complicating consistent inference. Importantly, the minor allele frequency of *BDNF* shows substantial variation across ethnic groups, underscoring the need for population-specific investigations to identify genetic risk factors relevant to Koreans [35,36].

We found that the Val66Met variant reduces BDNF protein levels, contributing to biological functions in energy metabolism connected with major synaptic functions in the brain [37]. Dooley et al. and Eyileten et al. reported that carriers of the Met allele have significantly lower serum BDNF concentrations compared with Val/Val carriers, which may in turn alter susceptibility to metabolic diseases [38,39]. We found BDNF is significantly increased when the carriers become obese with wild because biological functions of *BDNF*, such as satiety and responsiveness to food by hypothalamic signals, are increased for defense mechanisms. Conversely, Met carriers showed BDNF levels that were not changed in both non-OB and OB. It suggests that control of dietary restraint and the functional activity in brain regions associated with impulsive eating behaviors was impaired [40]. *BDNF* polymorphisms are believed to contribute to distinct disorders via different neurocognitive mechanisms depending on the eating disorder phenotype [41]. However, the biological interpretation of these findings should be made with caution, as peripheral BDNF concentrations do not necessarily reflect central nervous system activity.

In a Korean cohort study, Val66Met polymorphism was identified as a genetic risk factor significantly associated with BMI. Met allele carriers showed stronger correlations with smoking, and smokers tended to have lower BMI and BDNF levels [19]. Another Korean study examining the association between *BDNF* and type 2 diabetes found that Val/Met and Met/Met genotypes were inversely associated with diabetes risk and interacted with total energy and protein intake, suggesting gene–diet interactions [42]. We confirmed that the *BDNF* variant interacts with multiple indicators of obesity risk. In the BMI model, the negative association of RMR/BW suggests that decreased metabolic efficiency may contribute to an increase in BMI. Consistently, Gunstad et al. reported that *BDNF* is involved in energy homeostasis and lipid metabolism, rendering variant carriers more susceptible to metabolic imbalance [43,44]. Therefore, Met allele carriers may be more sensitive to weight gain. In previous work, RMR was shown to approximate ~60% of total energy requirements and is considered a highly heritable risk factor for obesity [21]. In this study, RMR/BW was lowest in A allele carriers in the OB group, indicating that energy expenditure efficiency was most impaired in obese carriers of the *BDNF* variant. These findings are consistent with animal studies in which hypothalamic administration of *BDNF* reduced food intake and increased energy expenditure, leading to weight loss, and suggest that the energy expenditure promoting effects of *BDNF* may be partially attenuated in the presence of the *rs6265* variant, resulting in weight gain and metabolic inefficiency [45]. In addition, in the RMR regression model, the age-related decline in RMR and higher RMR in men were in line with previous reports, and the relatively lower RMR observed in A allele carriers compared with the GG genotype supports the notion that A allele carriers may be more susceptible to weight gain because of the reduced basal metabolic rate [44,46,47]. In the WHR model, *BDNF* variants were positively associated with abdominal obesity, while RMR/BW was negatively correlated, implying that higher metabolic efficiency reduces fat accumulation. Zhang et al. proposed that WHR mediates the relationship between the *BDNF* genotype and serum BDNF concentrations [48]. The findings suggest that WHR may complement BMI as an indicator of the obesity phenotype [49]. While some studies have examined surrogate markers of metabolic syndrome such as WC, serum TG, HDLc, blood pressure, FBS, insulin resistance, leptin, and nutrient intake, the available evidence remains insufficient [50].

Regarding diets, A allele carriers were characterized by increased consumption of high-fat and high-protein foods, thereby elevating the risk of obesity with other metabolic disorders [51]. In our study, in risk variation (H + M), energy intakes did not increase, but energy expenditure (RMR/BW) decreased. Although some studies have reported that Vit A promotes BDNF expression and is consumed less by A allele carriers, our findings do not support this [52]. Mean Vit A intake in our cohort was 367.42 μg RAE, below the Korean recommended intake (600–800 μg RAE), suggesting the need for general increases in Vit A consumption. In our previous cohort study, we found high salt consumption is a big risk factor for obese children, particularly in the variation of salt-sensitive genes [53]. In this study, sodium consumption was higher in *BDNF* A allele carriers than in the GG type, and it was higher in A allele carriers with OB status than A allele carriers with non-OB. Although this trend was not statistically significant because of high SDs, it suggests that A allele carriers with OB may particularly benefit from sodium reduction as part of their dietary management. We concluded that sodium intake may suppress *BDNF* in OB status in case of A allele carriers and the defense mechanism being impaired. Shin et al. suggest that BDNF levels decreased or were aberrantly expressed in the cerebral cortex and hippocampus of fetuses in sodium-overloaded rats [54]. In other reports, adequate sodium and high sugar (>10% of energy intake) intakes showed higher BDNF levels (*p* < 0.05) [55]. In addition to sugar, foods with a high glycemic index such as refined sugar, white rice, instant potatoes and soda, should be prohibited for A allele carriers. Since it leads to insulin resistance, inflammation, cognitive decline, and impaired appetite, high HbA1c was found in our subjects with *BDNF* A allele carriers. The inflammation, oxidative stress, and alteration of BDNF were induced in fructose-fed rats, as well as an increase in glycation products. Interestingly, many of these alterations (*BDNF*, and dysregulation of neurotransmitters) persisted after switching to the control diet [56]. Therefore, extreme attention should be devoted to limiting excessive consumption of sweet foods that can affect brain physiology. It is well known that w-3 fatty acids are essential nutraceuticals for brain health, and that they produce neurobiological effects associated with prevention of depression. The Boston Puerto Rican study that showed interaction analyses between *BDNF* variations and diets suggests that PUFA consumption may explain these differences, although the cause of sex difference remains unclear [31]. Intake of PUFAs was shown to increase the production of *BDNF* in the brain, and the metabolites of the PUFAs have shown more binding affinity towards *BDNF* [57].

We found *BDNF rs6265* A allele carriers (GA + AA) had higher frequencies of alcohol consumption together with elevated levels of liver damage biomarkers (AST, ALT). Needs et al. found that the *BDNF* gene (*rs6265*/Val66Met) modulates the central nervous system of neurotransmitters, serving as a pathogenetic mechanism underlying alcohol use and it is critical for early development of alcohol addiction in healthy adolescents [58]. Colzato et al. reported that *BDNF* Met carriers were more anxious during the stress procedure (*p* < 0.001), drank more alcohol per week, (*p* < 0.05), and showed significantly higher anticipatory cortisol response (*p* < 0.05) than wild homozygote carriers [59]. These results suggest that Met carriers are particularly sensitive to anticipating stressful events and may engage in subsequent behaviors such as increased alcohol consumption. In the meta-analysis, smokers exhibited higher plasma levels of BDNF compared to non-smokers, although the potential therapeutic implications of BDNF for smoking were not elucidated [60]. Previous studies suggest that both drinking and smoking may be associated with *BDNF*. 

The limitations of this study can be summarized as follows. First, smaller genetic effects may have been underpowered, although the sample size was adequate to identify observed associations between the *BDNF* rs6265 genotype and obesity-related phenotypes. The relatively small sample size and imbalance across genotypes and sexes may have limited the statistical power and reduced the generalizability of the findings. Second, voluntary participation by individuals with high interest in weight loss and obesity-related genetic testing may have introduced selection bias, as their energy and nutrient intakes could differ from those of the general population, potentially distorting associations between dietary intake and phenotypic outcomes. Third, given the cross-sectional nature of the present study, the proposed recommendations for *BDNF* risk carriers should be interpreted with caution. Therefore, larger, well-powered studies—particularly longitudinal or genotype-specific intervention designs—are required to fully evaluate the effects of DTC-OB genes, and to propose precision nutrition strategies for genetic risk carriers in Korean populations. However, this study was designed to contribute scientific evidence that may inform evidence-based discussions among policymakers, industry, and consumers, rather than to support direct clinical or regulatory recommendations at this stage.

## 5. Conclusions

This is the first study to demonstrate that the *BDNF rs6265* polymorphism is significantly associated with obesity-related metabolic indicators and dietary patterns in Koreans. *BDNF rs6265* A allele carriers (GA + AA) as the obesity susceptibility allele exhibited lower metabolic efficiency and a less favorable metabolic profile compared to wild type carriers. These findings provide foundational evidence for the development of precision nutrition strategies tailored to *BDNF* variation-based personalized healthcare services. They may include moderation of dietary intake of sodium, Vit A, sugars, cholesterol and PUFA to support glycemic and lipid control; incorporating regular physical activity to improve RMR/BW; avoiding alcohol consumption to migrate liver-related metabolic risk. Further genetic-stratified intervention or longitudinal studies are warranted to validate these findings and to refine gene-based precision nutrition strategies for obesity prevention in Korean adults.

## Figures and Tables

**Table 1:** Anthropometric characteristics and biochemical markers by obesity status and sex.

Variables	Total (*n* = 231)	non-OB (*n* = 103)	OB (*n* = 128)	*p*-Value	*p*-Value ^adj^	Female (*n* = 200)	Male (*n* = 31)	*p*-Value	*p*-Value ^adj^
Anthropometric Characteristics									
Age (years)	30.93 ± 10.51	28.73 ± 9.24	32.70 ± 11.16	0.004 ^a)^ **	0.052	29.71 ± 9.87	38.81 ± 11.28	<0.001 ^a)^ ***	-
BMI (kg/m^2^)	24.21 ± 4.30	20.68 ± 1.60	27.05 ± 3.63	<0.001 ^b)^ ***	<0.001 ***	23.87 ± 4.19	26.42 ± 4.39	0.002 ^a)^ **	0.020 *
Ht (cm)	163.54 ± 7.20	162.61 ± 5.92	164.28 ± 8.03	0.070 ^b)^	0.691	161.77 ± 5.78	174.91 ± 4.66	<0.001 ^a)^ ***	<0.001 ***
Wt (kg)	64.98 ± 13.64	54.75 ± 5.92	73.21 ± 12.48	<0.001 ^b)^ ***	<0.001 ***	62.48 ± 11.57	81.08 ± 15.11	<0.001 ^a)^ ***	<0.001 ***
RMR (kcal)	1472.76 ± 190.30	1360.61 ± 86.09	1563.01 ± 203.03	<0.001 ^b)^ ***	<0.001 ***	1422.65 ± 117.06	1796.06 ± 248.99	<0.001 ^b)^ ***	<0.001 ***
RMR/BW	23.08 ± 2.41	25.01 ± 1.73	21.54 ± 1.65	<0.001 ^a)^ ***	<0.001 ***	23.19 ± 2.50	22.37 ± 1.61	0.018 ^b)^ *	0.692
WC (cm)	79.32 ± 11.62	70.77 ± 6.05	86.20 ± 10.40	<0.001 ^b)^ ***	<0.001 ***	77.42 ± 10.38	91.58 ± 11.89	<0.001 ^a)^ ***	<0.001 ***
WHR	0.80 ± 0.07	0.76 ± 0.05	0.83 ± 0.07	<0.001 ^b)^ ***	<0.001 ***	0.79 ± 0.06	0.87 ± 0.07	<0.001 ^a)^ ***	<0.001 ***
SBP (mmHg)	117.18 ± 13.46	113.52 ± 12.09	120.13 ± 13.83	<0.001 ^a)^ ***	0.010 **	115.18 ± 12.26	130.13 ± 13.90	<0.001 ^a)^ ***	<0.001 ***
DBP (mmHg)	70.48 ± 10.52	69.12 ± 9.68	71.57 ± 11.06	0.078 ^a)^	0.575	69.37 ± 9.90	77.65 ± 11.68	<0.001 ^a)^ ***	0.001 **
Biochemical Markers									
AST (IU/L)	18.48 ± 5.52	17.07 ± 4.02	19.62 ± 6.27	<0.001 ^b)^ ***	0.047 *	17.71 ± 4.87	23.45 ± 6.85	<0.001 ^b)^ ***	<0.001 ***
ALT (IU/L)	14.54 ± 9.35	10.69 ± 4.91	17.64 ± 10.82	<0.001 ^b)^ ***	<0.001 ***	13.16 ± 8.10	23.45 ± 11.83	<0.001 ^b)^ ***	<0.001 ***
AST/ALT	1.49 ± 0.46	1.73 ± 0.42	1.30 ± 0.41	<0.001 ^a)^ ***	<0.001 ***	1.54 ± 0.44	1.19 ± 0.50	<0.001 ^a)^ ***	0.004 **
FBS (mg/dL)	90.35 ± 15.4	88.82 ± 15.88	91.59 ± 14.95	0.173 ^a)^	0.833	88.73 ± 10.41	100.87 ± 31.09	0.039 ^b)^ *	<0.001 ***
HbA1c (%)	5.37 ± 0.39	5.29 ± 0.30	5.44 ± 0.43	0.003 ^a)^ **	0.153	5.32 ± 0.27	5.68 ± 0.72	0.011 ^b)^ *	<0.001 ***
TG (mg/dL)	107.30 ± 82.59	81.61 ± 44.25	127.98 ± 99.07	<0.001 ^b)^ ***	0.001 ***	98.91 ± 73.80	161.45 ± 112.56	<0.005 ^b)^ ***	0.004 **
TC (mg/dL)	192.98 ± 33.12	186.83 ± 29.31	197.92 ± 35.23	0.011 ^a)^ *	0.066	191.49 ± 31.38	202.61 ± 42.02	0.166 ^b)^ *	0.488
HDLc (mg/dL)	62.72 ± 15.40	70.41 ± 14.09	56.54 ± 13.55	<0.001 ^a)^ ***	<0.001 ***	64.2 ± 15.56	53.19 ± 10.19	<0.001 ^b)^ ***	0.003 **
LDLc (mg/dL)	116.74 ± 30.89	108.66 ± 25.73	123.24 ± 33.18	<0.001 ^b)^ ***	0.002 **	115.6 ± 29.53	124.1 ± 38.31	0.245 ^b)^	0.536
Leptin(ng/mL)	21.55 ± 15.44	14.94 ± 9.00	26.87 ± 17.40	<0.001 ^b)^ ***	<0.001 ***	23.14 ± 15.59	11.27 ± 9.46	<0.001 ^b)^ ***	<0.001 ***

Values are presented as means ± SDs, *p*-values were determined by Student’s *t*-test ^a)^ or Welch’s *t*-test ^b)^; * *p* < 0.05, ** *p* < 0.01, *** *p* < 0.001. Adjusted *p*-values were determined by ANCOVA. For comparisons by obesity status, *p*-values **^adj^** were adjusted for age and sex. For comparisons by sex, *p*-values were adjusted for age. Abbreviations: BMI, body mass index; Ht, height; Wt, weight; RMR, resting metabolic rate; RMR/BW, resting metabolic rate/body weight; WHR, waist-to-hip ratio; SBP, systolic blood pressure; DBP, diastolic blood pressure; AST, aspartate aminotransferase; ALT, alanine aminotransferase; FBS, fasting blood glucose; HbA1c, Hemoglobin A1c; TG, triglycerides; TC, total cholesterol; HDLc, high-density lipoprotein cholesterol; LDLc, low-density lipoprotein cholesterol.

**Table 2:** Comparison of genotype frequencies between the non-OB and OB groups according to genetic polymorphisms.

Gene	SNPs	Genotypes	Frequency			MAF	χ^2^	*p*-Value
			Total	non-OB	OB			
*FTO*	*rs9939609*	TT	174 (75.3)	80 (77.7)	94 (73.4)	13.0	2.637	0.268 ^a)^
		TA	54 (23.4)	23 (22.3)	31 (24.2)			
		AA	3 (1.3)	0 (0.0)	3 (2.3)			
		TT	174 (75.3)	80 (77.7)	94 (73.4)		0.550	0.540 ^b)^
		TA + AA	57 (24.7)	23 (22.3)	34 (26.6)			
	*rs9939973*	GG	161 (69.7)	75 (72.8)	86 (67.2)	16.2	1.728	0.422 ^a)^
		GA	65 (28.1)	27 (26.2)	38 (29.7)			
		AA	5 (2.2)	1 (1.0)	4 (3.1)			
		GG	161 (69.7)	75 (72.8)	86 (67.2)		0.856	0.389 ^b)^
		GA + AA	70 (30.3)	28 (27.2)	42 (32.8)			
	*rs8050136*	CC	174 (75.3)	80 (77.7)	94 (73.4)	13.0	2.637	0.268 ^a)^
		CA	54 (23.4)	23 (22.3)	31 (24.2)			
		AA	3 (1.3)	0 (0.0)	3 (2.3)			
		CC	174 (75.3)	80 (77.7)	94 (73.4)		0.550	0.540 ^b)^
		CA + AA	57 (24.7)	23 (22.3)	34 (26.6)			
*MC4R*	*rs17782313*	TT	122 (52.8)	48 (46.6)	74 (57.8)	27.1	3.979	0.137 ^a)^
		CT	93 (40.3)	45 (43.7)	48 (37.5)			
		CC	16 (6.9)	10 (9.7)	6 (4.7)			
		TT	122 (52.8)	48 (46.6)	74 (57.8)		2.878	0.112 ^b)^
		CT + CC	109 (47.2)	55 (53.4)	54 (42.2)			
*BDNF*	*rs6265*	GG	65 (28.1)	36 (35.0)	29 (22.7)	47.4	5.030	0.081 ^a)^
		GA	113 (48.9)	43 (41.7)	70 (54.7)			
		AA	53 (22.9)	24 (23.3)	29 (22.7)			
		GG	65 (28.1)	36 (35.0)	29 (22.7)		4.267	0.041 ^b)^ *
		GA + AA	166 (71.9)	67 (65.0)	99 (77.3)			

Values are presented as *n* (%), number of subjects (percentage). Group differences were determined using Pearson’s chi-square test ^a)^ or Fisher’s exact test ^b)^; * p < 0.05. Abbreviations: *FTO*, fat mass and obesity-associated gene; *MC4R*, melanocortin-4 receptor; *BDNF*, brain-derived neurotrophic factor.

**Table 3:** Anthropometric characteristics, biochemical markers, and dietary intake according to *BDNF* genotypes and obesity status.

Variables	Total	GG		GA + AA		*p*-Value	*p*-Value ^adj^
		non-OB (*n* = 36)	OB (*n* = 29)	non-OB (*n* = 67)	OB (*n* = 99)		
**Anthropometric Characteristics**							
BMI (kg/m^2^)	24.21 ± 4.3	21.03 ± 1.57	27.08 ± 3.81	20.49 ± 1.6	27.04 ± 3.6	0.560	0.723
SMM (kg)	23.65 ± 5.18	21.55 ± 1.77	25.07 ± 5.54	20.74 ± 3.16	25.78 ± 5.73	0.292	0.329
FM (kg)	22.04 ± 8.42	16.62 ± 4.24	27.46 ± 8.41	15.47 ± 3.7	26.46 ± 7.78	0.938	0.934
Wt (kg)	64.98 ± 13.64	56.23 ± 5.45	73.94 ± 13.97	53.95 ± 6.05	72.99 ± 12.07	0.657	0.706
WC (cm)	79.32 ± 11.62	71.01 ± 5.42	85.47 ± 9.83	70.64 ± 6.4	86.42 ± 10.59	0.611	0.932
WHR	0.8 ± 0.07	0.75 ± 0.04	0.81 ± 0.06	0.77 ± 0.06	0.83 ± 0.07	0.688	0.661
RMR (kcal)	1472.76 ± 190.3	1365.34 ± 80.94	1583.2 ± 227.47	1358.07 ± 89.23	1557.1 ± 196.16	0.695	0.583
RMR/BW	23.08 ± 2.41	24.39 ± 1.37	21.62 ± 1.63	25.34 ± 1.82	21.51 ± 1.67	0.034	0.386
SBP (mmHg)	117.18 ± 13.46	110.53 ± 11.07	121.17 ± 13.99	115.13 ± 12.39	119.82 ± 13.84	0.124	0.060
DBP (mmHg)	70.48 ± 10.52	67.08 ± 10.73	70.31 ± 11.55	70.21 ± 8.95	71.94 ± 10.95	0.628	0.313
**Clinical Markers**							
AST (IU/L)	18.48 ± 5.52	17.19 ± 4.21	17.97 ± 5.21	17 ± 3.94	20.1 ± 6.49	0.143	0.493
ALT (IU/L)	14.54 ± 9.35	10.11 ± 3.44	15.28 ± 7.55	11 ± 5.54	18.33 ± 11.55	0.399	0.738
AST/ALT	1.49 ± 0.46	1.78 ± 0.38	1.31 ± 0.35	1.71 ± 0.44	1.3 ± 0.43	0.649	0.357
FBS (mg/dL)	90.35 ± 15.4	88.78 ± 12.35	88.38 ± 8.81	88.84 ± 17.57	92.54 ± 16.23	0.368	0.591
HbA1c (%)	5.37 ± 0.39	5.27 ± 0.24	5.32 ± 0.18	5.3 ± 0.33	5.47 ± 0.48	0.269	0.681
TG (mg/dL)	107.3 ± 82.59	81.19 ± 45.21	127.03 ± 120.74	81.84 ± 44.07	128.25 ± 92.49	0.981	0.582
TC (mg/dL)	192.98 ± 33.12	191.14 ± 25.69	190.38 ± 26.93	184.52 ± 31.02	200.13 ± 37.14	0.091	0.236
HDLc (mg/dL)	62.72 ± 15.4	72.22 ± 13.42	56.76 ± 12.14	69.43 ± 14.44	56.47 ± 13.99	0.540	0.372
LDLc (mg/dL)	116.74 ± 30.89	111.33 ± 23.97	117.9 ± 29.08	107.22 ± 26.7	124.81 ± 34.27	0.217	0.381
Leptin(ng/mL)	21.55 ± 15.44	15.94 ± 9.89	30.88 ± 19.36	14.4 ± 8.51	25.69 ± 16.71	0.387	0.590
**Dietary intakes**							
Energy (kcal)	1798.28 ± 616.68	1816.75 ± 606.82	1755.07 ± 511.34	1718.67 ± 447.69	1857.3 ± 735.55	0.274	0.349
CHO (g)	212.17 ± 72.78	207.84 ± 73.09	207.14 ± 53.80	210.47 ± 62.34	216.34 ± 83.85	0.762	0.262
Fat (g)	64.88 ± 27.31	70.06 ± 30.37	63.87 ± 25.25	61.07 ± 21.15	65.83 ± 30.23	0.177	0.332
Protein (g)	76.75 ± 29.30	79.06 ± 27.32	74.83 ± 23.03	70.24 ± 22.95	80.82 ± 34.5	0.087	0.211
Fiber (g)	16.89 ± 8.25	17.39 ± 6.98	13.77 ± 5.02	15.39 ± 5.94	18.62 ± 10.18	0.005	0.050
Sugar (g)	37.78 ± 22.90	44.25 ± 23.58	34.29 ± 24.39	38.03 ± 22.65	36.28 ± 22.25	0.225	0.672
Vit A (RAE)	367.42 ± 284.01	366.92 ± 261.53	341.3 ± 162.95	326.07 ± 156.10	402.82 ± 371.07	0.224	0.730
Vit D (μg)	1.40 ± 1.17	1.55 ± 1.18	1.40 ± 1.24	1.28 ± 1.24	1.42 ± 1.11	0.397	0.842
Vit E (mg)	11.20 ± 6.62	12.04 ± 8.29	10.04 ± 4.32	9.39 ± 4.89	12.45 ± 7.23	0.009	0.050
Vit C (mg)	53.83 ± 40.49	50.88 ± 42.14	42.90 ± 33.47	51.43 ± 40.87	59.71 ± 41.12	0.174	0.605
Vit B1 (mg)	1.26 ± 1.19	1.54 ± 2.27	0.98 ± 0.31	1.22 ± 1.21	1.26 ± 0.62	0.086	0.101
Vit B2 (mg)	1.42 ± 0.57	1.46 ± 0.62	1.32 ± 0.43	1.34 ± 0.45	1.48 ± 0.64	0.095	0.345
Niacin(mg)	13.82 ± 6.26	13.64 ± 6.03	13.74 ± 5.38	13.14 ± 6.05	14.37 ± 6.74	0.549	0.904
Vit B6 (mg)	0.57 ± 0.60	0.65 ± 1.25	0.50 ± 0.31	0.54 ± 0.36	0.57 ± 0.41	0.314	0.422
Folate (μg)	210.87 ± 107.3	231.76 ± 140.44	175.41 ± 75.76	185.06 ± 71.15	230.86 ± 116.09	0.001	0.012
Vit B12(μg)	3.47 ± 2.43	3.34 ± 1.89	3.16 ± 2.13	3.46 ± 2.43	3.62 ± 2.69	0.640	0.719
Ca (mg)	457.38 ± 225.53	497.84 ± 209.97	430.82 ± 173.75	421.53 ± 191.34	474.35 ± 261.21	0.073	0.340
P (mg)	1018.45 ± 382.26	1035.6 ± 398.45	976.80 ± 303.66	917.71 ± 307.54	1091.57 ± 427.58	0.038	0.161
Na (mg)	3593 ± 1781.7	3128.41 ± 1336.6	3223.16 ± 1763.9	3454.89 ± 1375.7	3962.35 ± 2089.3	0.429	0.995
K (mg)	2200.85 ± 945.49	2185.79 ± 925.57	1918.64 ± 645.66	1976.17 ± 669.89	2438.77 ± 1118.66	0.008	0.074
Na/K	1.72 ± 0.69	1.55 ± 0.67	1.73 ± 0.79	1.82 ± 0.60	1.72 ± 0.72	0.171	0.397
Mg (mg)	198.63 ± 94.59	208.59 ± 115.27	175.87 ± 77.15	174.22 ± 69.26	217.96 ± 101.56	0.006	0.069
Fe (mg)	11.93 ± 7.57	12.77 ± 10.66	11.05 ± 5.01	11.81 ± 8.36	11.97 ± 6.26	0.403	0.651
Cholesterol (mg)	263.02 ± 138.3	270.96 ± 130.71	239.86 ± 111.47	233.53 ± 111.61	286.59 ± 159.54	0.039	0.085
FA (g)	48.39 ± 22.25	53.07 ± 26.89	46.83 ± 20.37	44.37 ± 17.46	49.82 ± 23.59	0.076	0.216
SFA (g)	18.48 ± 10.7	20.89 ± 11.09	19.05 ± 8.53	17.29 ± 10.58	18.24 ± 11.2	0.380	0.772
MUFA (g)	18.52 ± 9.29	20.11 ± 11.10	17.63 ± 8.32	17.19 ± 7.45	19.08 ± 9.91	0.113	0.380
PUFA (g)	12.8 ± 6.44	14.23 ± 7.92	11.86 ± 5.10	11.59 ± 5.36	13.36 ± 6.76	0.030	0.103
TFA (g)	0.45 ± 0.30	0.43 ± 0.28	0.52 ± 0.37	0.41 ± 0.27	0.47 ± 0.30	0.706	0.164

Values are presented as mean ± SD. Data were assessed by two-way ANOVA. *p*-Value ^adj^ represents *p*-values adjusted by ANCOVA for age, sex, and total energy intake. Abbreviations: SMM, skeletal muscle mass; FM, fat mass; Wt, weight; WC, waist circumference; WHR, waist-to-hip ratio; RMR, resting metabolic rate; RMR/BW, resting metabolic rate/body weight; SBP, systolic blood pressure; DBP, diastolic blood pressure; AST, aspartate aminotransferase; ALT, alanine aminotransferase; FBS, fasting blood glucose; HbA1c, hemoglobin A1c; TG, triglyceride; TC, total cholesterol; HDLc, high-density lipoprotein cholesterol; LDLc, low-density lipoprotein cholesterol; CHO, carbohydrate; Vit, vitamin; Ca, calcium; P, phosphorus; Na, sodium; K, potassium; Mg, magnesium; Fe, Iron; FA, fatty acid; SFA, saturated fatty acid; MUFA, mono-unsaturated fatty acid; PUFA, poly-unsaturated fatty acid; TFA, trans fatty acid.

**Table 4:** Factors contributing to increased obesity risk using obesity-related dependent variables.

Obesity Phenotypes	Variables	β (95% CI)	*p*-Value ^a)^	*p*-Value ^adj^	F	*p-* Value ^b)^	R^2^	Adj. R^2^
BMI	*BDNF*	0.051 (0.038, 0.932)	0.033 *	0.053	208.102	<0.001	0.883	0.879
	Sex	−0.147 (−2.867, −0.824)	<0.001 ***	0.008				
	RMR (kcal)	0.417 (0.007, 0.011)	<0.001 ***	0.004				
	RMR/BW	−0.465 (−0.957, −0.702)	<0.001 ***	0.003				
	WHR	0.067 (0.245, 8.333)	0.038 *	0.051				
	ALT (IU/L)	0.058 (0.000, 0.053)	0.047 *	0.047				
	HDLc (mg/dL)	−0.055 (−0.030, 0.000)	0.044 *	0.050				
	Leptin (ng/mL)	0.207 (0.041, 0.074)	<0.001 ***	0.002				
RMR	*BDNF*	−0.074 (−59.624, −2.827)	0.031 *	0.031	96.643	<0.001	0.754	0.746
	Sex	0.671 (327.340, 420.160)	<0.001 ***	<0.001				
	Age	−0.311 (−6.977, −4.256)	<0.001 ***	<0.001				
	WHR	0.158 (198.358, 690.985)	<0.001 ***	<0.001				
	SBP (mmHg)	0.128 (0.753, 2.870)	<0.001 ***	<0.001				
	ALT (IU/L)	0.186 (2.136, 5.422)	<0.001***	<0.001				
	Leptin (ng/mL)	0.317 (2.985, 4.809)	<0.001 ***	<0.001				
WHR	*BDNF*	0.100 (0.001, 0.029)	0.036 *	0.043	39.512	<0.001	0.516	0.503
	Sex	0.251 (0.029, 0.070)	<0.001 ***	0.006				
	RMR/BW	−0.415 (−0.015, −0.009)	<0.001 ***	0.003				
	ALT (IU/L)	0.202 (0.001, 0.002)	<0.001 ***	0.002				
	Vit A (μg RAE)	0.184 (−6 × 10^−4^, 3 × 10^−5^)	<0.001 ***	0.002				
	Sugar (g)	−0.086 (−1 × 10^−3^, 1 × 10^−4^)	0.076	0.076				

Regression coefficients (β) are presented with 95% confidence intervals (CI). Reference group: sex, female; *BDNF* genotype, wild. * *p* < 0.05, *** *p* < 0.001. *p*-values ^a)^ for each predictor, overall model F-statistics, *p*-values ^b)^ for the regression models, and coefficients of determination (R^2^, adjusted R^2^) are presented. Adjusted *p*-values (*p*-values ^adj^ were calculated using the Benjamini–Hochberg false discovery rate (FDR) procedure to account for multiple comparisons). Abbreviations: BMI, body mass index; WHR, waist-to-hip ratio; RMR, resting metabolic rate; RMR/BW, resting metabolic rate/body weight; SBP, systolic blood pressure; ALT, alanine aminotransferase; HDLc, high-density lipoprotein cholesterol.

## Data Availability

The data presented in this study are available on request from the corresponding author due to privacy and ethical restrictions.

## Supplementary Materials

The following supporting information can be downloaded at: https://www.mdpi.com/article/10.3390/nu18040655/s1, Table S1: Ethnic differences in minor allele frequencies (MAF) of *FTO*, *MC4R*, and *BDNF Polymorphisms*., Table S2: TaqMan assay, primer sequences, and PCR conditions used for SNP genotyping., Table S3: Sociodemographic, dietary, and health behavioral characteristics by obesity status and sex., Table S4: Pearson correlation matrix of predictors used in regression models to assess multicollinearity., Table S5: Variance inflation factors (VIF) and tolerance values for multicollinearity assessment in regression models.

## Abbreviations

ALT, alanine aminotransferase; AST, aspartate aminotransferase; BIA, bioelectrical impedance analysis; BDNF, brain-derived neurotrophic factor; BMI, body mass index; Ca, calcium; CHO, carbohydrate; DBP, diastolic blood pressure DTC, direct-to-consumer; FA, fatty acid; FBS, fasting blood glucose; Fe, Iron; FM, fat mass; FTO, fat mass and obesity-associated gene; MC4R, melanocortin-4 receptor; FDR, false discovery rate; GWAS, genome wide association study; GIPR, gastric inhibitory polypeptide receptor; HbA1c, hemoglobin A1c; HC, hip circumference; Ht, height; HDLc, high-density lipoprotein cholesterol; IRB, Institutional Review Board; K, potassium; KGP 4K, Korean Genome Project 4K; KoGES, Korean Genome and Epidemiology Study; KRGDB, Korean Reference Genome Database; LDLc, low-density lipoprotein cholesterol; MAF, minor allele frequency; Mg, magnesium; MUFA, mono-unsaturated fatty acid; Na, sodium; NCBI, National Center for Biotechnology Information; NRXN3, neurexin 3; P, phosphorus; PUFA, polyunsaturated fatty acid; RMR, resting metabolic rate; RMR/BW, resting metabolic rate/body weight; SBP, systolic blood pressure; SEC16B, SEC16 homolog B endoplasmic reticulum export factor; SFA, saturated fatty acid; SMM, skeletal muscle mass; SNP, single nucleotide polymorphism; TC, total cholesterol; TFA, trans fatty acid; TG, triglyceride; Vit, vitamin; VIF, variance inflation factors; WC, waist circumference;. WHR, waist-to-hip ratio; Wt, weight.
