# Supplementary material for: Exploring the Association Between DTC Obesity-Related Gene Polymorphisms and Obesity Risk Factors in Koreans: Focus on BDNF
Source: Nutrients. 2026 Feb 16;18(4):655. doi: 10.3390/nu18040655 (PMC12942998; doi:10.3390/nu18040655)
Supplement: Supplementary file 1 [file nutrients-18-00655-s001.zip › nutrients-4118312-supplementary.pdf]

Supplementary Table S1. Ethnic differences in minor allele frequencies (MAF) of *FTO*, *MC4R*, and *BDNF* Polymorphisms.

	Gene	*FTO*			*MC4R*	*BDNF*
	SNPs	*rs 9939609*	*rs 9939973*	*rs 8050136*	*rs 17782313*	*rs 6265*
	Ref Allele	T	G	C	T	G(Val)
	Risk Allele	A	A	A	C	A(Met)
Korean	Total (Korean)[1)]	13.1	15.4	12.7	25.2	39.2
	This study	13.0	16.2	13.0	27.1	47.4
	Previous study[2)]	13.6	12.5	12.5	24.6	19.8
	KGP 4K	12.7	16.1	12.4	25.5	44.7
	KRGDB	13.1	16.7	13.0	23.7	44.8
Others[3)]	Total (Global)	39.9	42.8	39.7	23.0	18.9
	European	41.0	43.9	40.9	23.1	19.4
	African	47.4	42.5	43.6	28.4	4.2
	African Others	54.8	42.7	46.9	30.9	1.3
	African American	47.1	42.5	43.5	28.3	4.3
	Asian	13.0	16.9	13.0	20.4	44.5
	East Asian	12.5	16.7	12.5	21.8	45.4
	South Asian	28.7	40.6	32.6	39.6	19.1
	Other Asian	15.3	17.9	14.1	16.9	41.1
	Latin American 1	42.7	42.8	40.6	23.0	14.8
	Latin American 2	25.3	29.1	24.6	12.7	16.7

"-" indicates data not available.

1) Calculated by average Korean data.

2) Data extracted from previous studies in Korean populations. *FTO* [13–16]; *MC4R* [17,18]; *BDNF* [19].

3) MAFs of each SNP were retrieved from the NCBI dbSNP database.

Latin American 1: Latin American individiuals with Afro-Caribbean ancestry.

Latin American 2: Latin American individiuals with mostly European and Native American Ancestry.

Abbreviation: KGP 4K: Korean Genome Project 4K; KRGDB: Korean Reference Genome Database; *FTO*: fat mass and obesity associated gene; *MC4R*: melanocortin-4 Receptor; *BDNF*: brain-derived neurotrophic factor.

Supplementary Table S2. TaqMan assay, primer sequences, and PCR conditions used for SNP genotyping.

**Taqman probe type & assay ID**				
Gene	*rs* number	Conc.	Assay ID	Type
FTO	*rs9939609*	40X	C_30090620_10	Functionally Tested
Primer sequence & PCR condition (SNaPshot)				
Gene	*rs* number	Strand	Primersequence	
FTO	*rs9939973*	Forward	Forward Primer	ttacaggtgtgagcctctg
			Reverse Primer	ggctttaacccatcaccta
			Genotyping Primer	gcaccYaaggRaccatcaRagag
FTO	*rs8050136*	Forward	Forward Primer	GCCAGCTTCATAGCCTAGT
			Reverse Primer	gaccttggacaaaccactt
			Genotyping Primer	CAGTYGCCCACTKTGGCAAT
MC4R	*rs17782313*	Forward	Forward Primer	TGATGACCATCCTTTTCCTT
			Reverse Primer	CACTGGGAGTTCCAATGC
			Genotyping Primer	GCTGACACTTTCGAACAC
BDNF	*rs6265*	Forward	Forward Primer	GGATAGGGGAAACATGAAA
			Reverse Primer	AATCCCAGATGCTAAAATGA
			Genotyping Primer	TTTAAAGCAGGAGAGATTGTATCC

Abbreviation: Conc: concentration; *FTO*: fat mass and obesity associated gene; *MC4R*: melanocortin-4 Receptor; *BDNF*: brain-derived neurotrophic factor.

Supplementary Table S3. Sociodemographic, dietary, and health behavioral characteristics by obesity status and sex.

Variables	Total	OB		*p*-Value	Sex		*p*-Value	Genotypes		*p*-Value
		non-OB (n=103)	OB (n=128)		Female (n=200)	Male (n=31)		GG (n=65)	GA+AA (n=166)	
Sociodemographic Characteristics										
Education Level										
<High school	1(0.4)	0(0.0)	1(0.8)	0.086[a)]	0(0.0)	1(3.2)	0.034[a)*]	0(0.0)	1(0.6)	0.452[a)]
High school	62(27.2)	34(34.0)	28(21.9)		57(28.9)	5(16.1)		14(21.5)	48(29.4)	
College/University	126(55.3)	54(54.0)	72(56.3)		108(54.8)	18(58.1)		41(63.1)	85(52.1)	
Graduate degreee[†]	39(17.1)	12(12.0)	27(21.1)		32(16.2)	7(22.6)		10(15.4)	29(17.8)	
Economic Income[‡]										
<1 million	10(4.5)	7(7.2)	3(2.4)	0.326[a)]	10(5.2)	0(0.0)	0.603[a)]	4(6.3)	6(3.8)	0.597[a)]
1-2.99 million	25(11.2)	9(9.3)	16(12.7)		22(11.5)	3(9.7)		9(14.3)	16(10.0)	
3-4.99 million	59(26.5)	25(25.8)	34(27.0)		50(26.0)	9(29.0)		17(27.0)	42(26.3)	
≥5 million	129(57.8)	56(57.7)	73(57.9)		110(57.3)	19(61.3)		33(52.4)	96(60.0)	
Family History										
OB										
No	136(59.1)	71(69.6)	65(50.8)	0.005[b)**]	113(56.8)	23(74.2)	0.078[b)]	36(55.4)	100(60.6)	0.552[b)]
Yes	94(40.9)	31(30.4)	63(49.2)		86(43.2)	8(25.8)		29(44.6)	65(39.4)	
CVD										
No	92(39.8)	43(41.7)	49(38.3)	0.685[b)]	77(38.5)	15(48.4)	0.327[b)]	26(40.0)	66(39.8)	1.000[b)]
Yes	139(60.2)	60(58.3)	79(61.7)		123(61.5)	16(51.6)		39(60.0)	100(60.2)	
DB										
No	126(54.8)	59(57.8)	67(52.3)	0.426[b)]	106(53.3)	20(64.5)	0.332[b)]	34(52.3)	92(55.8)	0.661[b)]
Yes	104(45.2)	43(42.2)	61(47.7)		93(46.7)	11(35.5)		31(47.7)	73(44.2)	
Dietary Habits										
Skipping Breakfast										
None	22(9.6)	11(10.8)	11(8.6)	0.779[a)]	15(7.5)	7(22.6)	0.044[a)*]	6(9.2)	16(9.7)	0.741[a)]
1~2days	31(13.5)	11(10.8)	20(15.6)		29(14.6)	2(6.5)		9(13.8)	22(13.3)	
3~4days	42(18.3)	19(18.6)	23(18.0)		38(19.1)	4(12.9)		13(20.0)	29(17.6)	
5~6days	71(30.9)	34(33.3)	37(28.9)		64(32.2)	7(22.6)		16(24.6)	55(33.3)	
all days	64(27.8)	27(26.5)	37(28.9)		53(26.6)	11(35.5)		21(32.3)	43(26.1)	
Eating out for Lunch										

0~2days	142(64.0)	64(66.0)	78(62.4)	0.673[b)]	128(66.7)	14(46.7)	0.041[b)*]	38(63.3)	104(64.2)	1.000[b)]
3~7days	80(36.0)	33(34.0)	47(37.6)		64(33.3)	16(53.3)		22(36.7)	58(35.8)	
Eating out for Dinner										
0~2days	132(59.7)	58(58.6)	74(60.7)	0.784[b)]	117(61.3)	15(50.0)	0.317[b)]	38(60.3)	94(59.5)	1.000[b)]
3~7days	89(40.3)	41(41.4)	48(39.3)		74(38.7)	15(50.0)		25(39.7)	64(40.5)	
Health Behaviors										
Smoking										
No	200(87.3)	92(91.1)	108(84.4)	0.162[b)]	184(92.9)	16(51.6)	<0.001[b)***]	58(89.2)	142(86.6)	0.665[b)]
Yes	29(12.7)	9(8.9)	20(15.6)		14(7.1)	15(48.4)		7(10.8)	22(13.4)	
Alcohol Consumption										
None	99(43.0)	44(43.1)	55(43.0)	0.987[a)]	94(47.2)	5(16.1)	<0.001[a)***]	30(46.2)	69(41.8)	0.082[a)]
1~2days	98(42.6)	43(42.2)	55(43.0)		84(42.2)	14(45.2)		31(47.7)	67(40.6)	
3~7days	33(14.3)	15(14.7)	18(14.1)		21(10.6)	12(38.7)		4(6.2)	29(17.6)	
Sleeping time										
≤5h	22(9.6)	10(9.8)	12(9.4)	0.430[a)]	17(8.5)	5(16.1)	0.192[a)]	8(12.3)	14(8.5)	0.572[a)]
6-7h	152(66.1)	65(63.7)	87(68.0)		132(66.3)	20(64.5)		44(67.7)	108(65.5)	
8-9h	54(23.5)	25(24.5)	29(22.7)		49(24.6)	5(16.1)		13(20.0)	41(24.8)	
≥10h	2(0.9)	2(2.0)	0(0.0)		1(0.5)	1(3.2)		0(0.0)	2(1.2)	
Exercise										
Aerobic-Moderate Intensity										
None	60(26.2)	26(25.5)	34(26.8)	0.647[a)]	51(25.8)	9(29.0)	0.579[a)]	17(26.2)	43(26.2)	0.999[a)]
1~2days	91(39.7)	38(37.3)	53(41.7)		77(38.9)	14(45.2)		26(40.0)	65(39.6)	
≥3days	78(34.1)	38(37.3)	40(31.5)		70(35.4)	8(25.8)		22(33.8)	56(34.1)	
Aerobic-High Intensity										
None	124(53.9)	56(54.9)	68(53.1)	0.465[a)]	110(55.3)	14(45.2)	0.124[a)]	32(49.2)	92(55.8)	0.655[a)]
1~2days	69(30.0)	27(26.5)	42(32.8)		55(27.6)	14(45.2)		21(32.3)	48(29.1)	
≥3days	37(16.1)	19(18.6)	18(14.1)		34(17.1)	3(9.7)		12(18.5)	25(15.2)	
Weight Training										
None	133(58.3)	60(59.4)	73(57.5)	0.631[a)]	119(60.4)	14(45.2)	0.199[a)]	34(52.3)	99(60.7)	0.594[a)]
1~2days	60(26.3)	24(23.8)	36(28.3)		47(23.9)	13(41.9)		20(30.8)	40(24.5)	
≥3days	35(15.4)	17(16.8)	18(14.2)		31(15.7)	4(12.9)		11(16.9)	24(14.7)	

Sample sizes vary across variables due to missing data. Values are presented as n (%); number of subjects (percentage).
Group differences were determined using Pearson's chi-square test[a)] or Fisher's exact test[b)]; *p<0.05, **p<0.01, ***p<0.001.
†; Individuals with master's, doctoral, or Ph.D. degree.
‡; Refers to KRW, the official currency of the Republic of Korea.
Abbreviation: OB, obesity; CVD, cardiovascular disease; DB, diabetes mellitus.

Supplementary Table S4. Pearson correlation matrix of predictors used in regression models to assess multicollinearity.

	1	2	3	4	5	6	7	8
BMI Variables								
1. *BDNF*	1							
2. Sex	.135*	1						
3. RMR (kcal)	.034	.671***	1					
4. RMR/BW	-.018	-.119	-.529***	1				
5. WHR	.173**	.423***	.482***	-.548***	1			
6. ALT (IU/L)	.143*	.376***	.491***	-.444***	.519***	1		
7. HDLc (mg/dL)	-.106	-.245***	-.381***	.461***	-.427***	-.358***	1	
8. Leptin (ng/mL)	-.043	-.262***	.242***	-.496***	.124	.139*	-.231***	1
RMR Variables								
1. *BDNF*	1							
2. Sex	.135*	1						
3. Age	.116	.296***	1					
4. WHR	.173**	.423***	.417***	1				
5. SBP (mmHg)	.089	.379***	.194**	.364***	1			
6. ALT (IU/L)	.143*	.376***	.321***	.519***	.289***	1		
7. Leptin (ng/mL)	-.043	-.262***	-.138*	.124	.078	.139*	1	
WHR Variables								
1. *BDNF*	1							
2. Sex	.135*	1						
3. RMR/BW	-.018	-.119	1					
4. ALT (IU/L)	.143*	.376***	-.444***	1				
5. Vit A (µg RAE)	.026	.086	-.060	.064	1			
6. Sugar (g)	-.068	-.209**	.030	-.123	.056	1		

Values represent Pearson's correlation coefficients (r). No pairwise correlations exceeded |r| = 0.8, indicating no serious multicollinearity.; **p*<0.05, ***p*<0.01, ****p*<0.001.

Abbreviation: BMI, body mass index; RMR, resting metabolic rate; RMR/BW, resting metabolic rate/body weight; WHR, waist-to-hip ratio; SBP, systolic blood pressure; ALT, alanine aminotransferase; HDLc, high-density lipoprotein cholesterol; Vit A, vitamin A.

Supplementary Table S5. Variance inflation factors (VIF) and tolerance values for multicollinearity assessment in regression models.

	Tolerance	VIF
BMI		
BDNF	.935	1.069
Sex	.311	3.215
RMR (kcal)	.288	3.473
RMR/BW	.403	2.481
WHR	.509	1.965
ALT (IU/L)	.632	1.581
HDLc (mg/dL)	.719	1.391
Leptin (ng/mL)	.551	1.816
RMR		
BDNF	.957	1.045
Sex	.622	1.607
Age	.764	1.309
WHR	.567	1.764
SBP (mmHg)	.777	1.286
ALT (IU/L)	.664	1.506
Leptin (ng/mL)	.789	1.268
WHR		
BDNF	.967	1.034
Sex	.819	1.221
RMR/BW	.795	1.257
ALT (IU/L)	.685	1.459
Vit A (μg RAE)	.984	1.017
Sugar (g)	.947	1.056

Tolerance and variance inflation factor (VIF) values are presented for independent variables included in the regression models. All VIF values were <10 and tolerance values were >0.1, indicating no serious multicollinearity.

Abbreviation: BMI, body mass index; RMR, resting metabolic rate; RMR/BW, resting metabolic rate/body weight; WHR, waist-to-hip ratio; SBP, systolic blood pressure; ALT, alanine aminotransferase; HDLc, high-density lipoprotein cholesterol; Vit A, vitamin A.
